# Targeted next-generation sequencing provides novel clues for associated epilepsy and cardiac conduction disorder/SUDEP

**DOI:** 10.1371/journal.pone.0189618

**Published:** 2017-12-19

**Authors:** Monica Coll, Pasquale Striano, Carles Ferrer-Costa, Oscar Campuzano, Jesús Matés, Bernat del Olmo, Anna Iglesias, Alexandra Pérez-Serra, Irene Mademont, Ferran Picó, Antonio Oliva, Ramon Brugada

**Affiliations:** 1 Cardiovascular Genetics Center, IDIBGI, Dr. Trueta University Hospital, Parc Hospitalari Martí i Julià, Edifici, Salt (Spain); 2 Pediatric Neurology and Muscular Diseases Unit, Department of Neurosciences, Rehabilitation, Ophthalmology, Genetics, Maternal and Child Health, University of Genoa, "G. Gaslini" Institute, Genova (Italy); 3 Gendiag SL, Barcelona (Spain); 4 Department of Medical Sciences, School of medicine, University of Girona, Girona (Spain); 5 Centro de Investigación Biomédica en Red de Enfermedades Cardiovasculares (CIBERCV), Madrid (Spain); 6 Institute of Public Health, Section of Legal Medicine, Catholic University, Rome (Italy); 7 Cardiac Genetics Unit, Cardiology Service, Hospital Josep Trueta, Girona (Spain); Indiana University, UNITED STATES

## Abstract

Sudden unexpected death in epilepsy is an unpredicted condition in patients with a diagnosis of epilepsy, and autopsy does not conclusively identify cause of death. Although the pathophysiological mechanisms that underlie this entity remain unknown, the fact that epilepsy can affect cardiac function is not surprising. The genetic factors involving ion channels co-expressed in the heart and brain and other candidate genes have been previously described. In the present study, 20 epilepsy patients with personal or family history of heart rhythm disturbance/cardiac arrhythmias/sudden death were sequenced using a custom re-sequencing panel. Twenty-six relatives were genetically analysed to ascertain the family segregation in ten individuals. Four subjects revealed variants with positive genotype-phenotype segregation: four missense variants in the *CDKL5*, *CNTNAP2*, *GRIN2A* and *ADGRV1* genes and one copy number variant in *KCNQ1*. The potential pathogenic role of variants in new candidate genes will need further studies in larger cohorts, and the evaluation of the potential pathogenic role in the cardio-cerebral mechanisms requires in vivo/in vitro studies. In addition to family segregation, evaluation of the potential pathogenic roles of these variants in cardio-cerebral mechanisms by in vivo/in vitro studies should also be performed. The potential pathogenic role of variants in new candidate genes will need further studies in larger cohorts.

## Introduction

Sudden unexpected death (SUDEP) is the sudden, unexpected, witnessed or unwitnessed, non-traumatic, and non-drowning death in patients with epilepsy, with or without evidence of a seizure, with exclusion of documented status epilepticus, and no conclusive cause of death after a comprehensive medico-legal autopsy[1]. SUDEP is the most common cause of death in individuals with epilepsy and accounts for 7.5–17% of all deaths[2]. Nevertheless, the incidence of SUDEP varies widely due to the heterogeneity of the studies (type of epilepsy, size of studied cohort, definition of SUDEP and how the cause of death was determined)[3]. The risk of SUDEP is higher in males than in females; however, other risk factors, such as frequency of tonic-clonic seizures, early age of onset of epilepsy and polytherapy with antiepileptic drugs, have been identified as risk factors[1, 4]. The pathophysiological mechanisms of SUDEP are likely multifactorial, but it has been suggested that a specific genetic background may predispose these patients to potentially fatal cardiorespiratory dysfunctions[3]. Indeed, seizures may induce a variety of transient cardiac effects, which include arrhythmias, asystole and other heart abnormalities, some of which are potentially lethal[3]. As pathogenic abnormalities in genes co-expressed in the heart and in the brain may eventually result in SUDEP, several studies have described pathogenic alterations in genes encoding ion channels in patients with concomitant heart conduction disorder and SUDEP[5, 6].

The advent of massive parallel DNA sequencing platforms, referred to as next-generation sequencing (NGS) technology, has revolutionized the field of medical genomics, enabling fast and cost-effective generation of genetic data. We previously performed a genetic study in a large cohort of individuals with epilepsy and cardiac conduction disorders and showed putative pathogenic disease-causing alterations in genes encoding cardiac ion channels in approximately 25% of unrelated individuals [7]. However, since SUDEP likely involves some combination of abnormal brain and cardiac physiology, certain additional gene categories, e.g., encoding for proteins with a key function of cell excitability, could be suitable candidates to explain the physiopathology of SUDEP [8].

To test this hypothesis, we used applied NGS analysis to a follow-up cohort of patients with epilepsy associated with cardiac conduction disorder/SUDEP.

## Results

### Genetic findings

**Fig 1:**
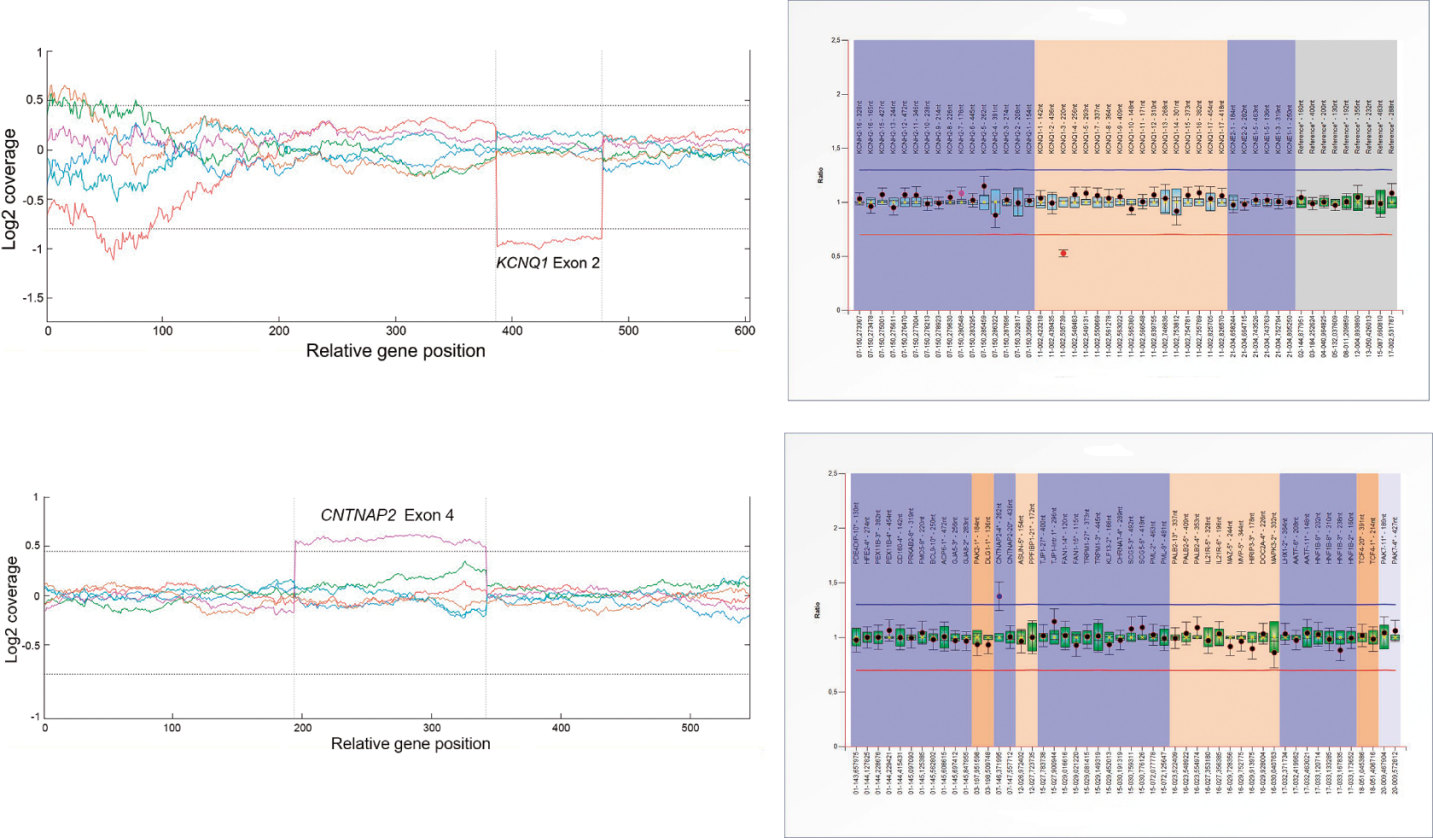
A: Deletion of exon 2 of the *KCNQ1* gene in ID#1. B: Duplication of exon 4 of the *CNTNAP2* gene in ID#15.

Nineteen out of the 20 recruited patients carried 56 rare genetic variants in 37 genes. Among these genetic anomalies, only four variants, i.e., *KCNQ1*, *KCNE1*, *SCN1A*, and *CACNA1C*, were previously associated with SUDEP or other disorders associated with sudden cardiac death (SCD). We also identified 2 CNVs (Fig 1), 45 missense variants, 6 indels and 3 intronic variants (Table 1).

**Table 1:** Rare genetic variants identified in our cohort.

Patient ID	Gene	Protein/cDNA variant	Variant Code	MAF (All)	*In silico*	Segregation	Pathogenicity Score
**Positive segregation**							
1^a^	*KCNQ1*	exon 2 deletion	-	-	-	Yes	Likely pathogenic
	*KCNE1*	p.Arg98Trp (c.292C>T)	rs199473362, CM002283	1.653x10^-5^	PD/D/DC	No	Benign
	*HTR2A*	p.Ile206Val (c.616A>G)	-	-	N/DC/DC	No	Benign
	*SENP2*	p.Ser231Cysfs*43 (c.692-693delCT)	-	-	-	No	Benign
	*CDKL5*	p.Arg914Lys (c.2741G>A*)	-	-	B/N/P	Yes	VUS
	*CACNA1H*	p.Thr78Ala (c.232A>G)	-	-	B/N/P	No	Benign
2	*CNTNAP2*	p.Ser339Asn (c.1016G>A)	-	-	N/DC/DC	Yes	VUS
3^a^	*GRIN2A*	p.Asn1076Lys (c.3228C>A)	rs61758995	0.004349	PD/N/DC	Yes	Likely non-pathogenic
	*CACNA1A*	p.His2216_His2217insHis (c.ins6646_6648insCAC)	-	-	-	IP	VUS
	*ADSL*	p.Asp430Asn (c.1288G>A)	rs554254383, CM001054	1.647x10^-5^		IP	VUS
	*ADGRV1*	p.Met1179Leu (c.3535A>T)	rs759416461	8.292x10^-6^	B/N/P	IP	Likely non-pathogenic
4^a^	*PLCB1*	p.Ala10Ser (c.28G>T)	rs150241349	0.0003402	PD/N/DC	No	Benign
	*ADGRV1*	p.Glu3215Lys (c.9643G>A)	rs199499672	0.0003396	PD/D/DC	Yes	VUS
**Incomplete penetrance**							
5^a^	*TSC1*	p.Ser108Phe (c.323C>T)	-	-	PD/D/DC	IP	VUS
6	*CELSR1*	p.Ala16_Ala17insAla (c.46_48insGCC)	-	-	-	No	Likely non-pathogenic
	*KCNQ2*	p.Val450Met (c.1348G>A)	rs146492238	0.001265	B/N/P	IP	VUS
	*HTR5A*	p.Pro95Leu (c.284C>T)	rs144847277	8.355x10-6	PD/D/DC	No	Likely non-pathogenic
	*SCN1A*	p.Arg1334Met (c.4001G>T)	-	-	PD/D/DC	IP	Likely pathogenic
7	*ADGRV1*	p.Arg4770His (c.14309G>A)	-	-	B/N/P	IP	Likely non-pathogenic
8^a^	*BRD2*	p.Ala599Pro (c.1795G>C)	rs55952113	0.004153	B/N/P	IP	Likely non-pathogenic
	*NRXN1*	c.1278+5A>T	rs201802152	0.002269	-	IP	Likely non-pathogenic
	*SCARB2*	p.Glu93Lys (c.277G>A)	rs145870223	0.0001573	PD/D/DC	No	Benign
	*POLG*	p.Lys512Arg (c.1535A>G)	-	-	B/N/P	No	Benign
	*HTR3B*	p.Ser156Arg (c.466A>C)	rs72466469, CM083528	0.004127	PD/N/DC	No	Benign
	*SCN1A*	p.Arg1928Gly (c.5782C>G)	rs121917956, CM081420	0.001427	B/N/DC	No	Benign
**No available relatives**							
9	*CACNA1H*	p.Met2312Val (c.6934A>G)	-	0.001677	B/N/P	NA	VUS
	*SLC2A1*	p.Arg400Cys (c.1198C>T)	-	-	PD/D/DC	NA	VUS
	*ADGRV1*	p.Thr879Met (c.2636C>T)	rs201007778	0.0001631	PD/D/DC	NA	Likely non-pathogenic
	*MBD5*	p.Pro721Leu (c.2162C>T)	rs138639760	0.0001982	B/N/DC	NA	Likely non-pathogenic
	*SCN2A*	c.605+3A>G	rs778554811	-	-	NA	VUS
10	*CACNA1C*	p.Val1707Ile (c.5119G>A)	rs147896322	-	PD/N/DC	NA	VUS
	*HTR2A*	p.Ala447Val (c.1340C>T)	rs6308, CM115490	0.001484	B/N/P	NA	Likely non-pathogenic
	*PNKP*	c.1029+2T>C	rs199919568	0.0006509	-	NA	Likely non-pathogenic
	*CACNA1I*	p.Asp2040Tyr (c.6118G>T)	rs771105041	0.004771	PD/D/P	NA	Likely non-pathogenic
11	*HCN2*	c.2143_2145delCCG	rs527536363	-	-	NA	VUS
	*CACNA1H*	p.Ala1570Val (c.4709C>T)	rs558718048	5.014x10-5	PD/D/DC	NA	VUS
12	*ADGRV1*	p.Pro4181Leu (c.12542C>T)	rs200957385	-	B/D/DC	NA	Likely non-pathogenic
	*KCNQ3*	p.Pro574Ser (c.1720C>T)	rs74582884, CM083709	0.001995	PD/N/DC	NA	Likely pathogenic
13	*CACNA1A*	p.2319Gln_2320GlninsGln (c.6955_6957insCAG)	-	-	-	NA	VUS
	*BRD2*	p.Asn194Lys (c.582C>G)	rs201572264	7.753x10-5	B/N/DC	NA	Likely non-pathogenic
	*ADGRV1*	p.Val754Ala (c.2261T>C)	rs374609813	-	PD/N/DC	NA	Likely non-pathogenic
	*HTR3B*	p.Gln334Glu (c.1001G>A)	rs199833563	8.238x10-6	B/N/P	NA	Likely non-pathogenic
14	*CACNA1A*	p.Gln2326_Gln2331del (c.6976_6993del18)	rs765169827	-	-	NA	VUS
	*SCARB2*	p.Val149Met (c.445G>A)	rs147159813	0.002053	B/N/P	NA	Likely non-pathogenic
	*CNTNAP2*	exon 4 duplication	-	-	-	NA	VUS
15	*CACNA1I*	p.Arg1448Gln (c.4343G>A)	rs201480163	9.944x10-5	PD/D/DC	NA	VUS
16	*GRIN2B*	p.Lys1090Arg (c.3269A>G)	-	-	PD/D/DC	NA	VUS
	*HTR7*	p.Lys445Asn (c.1335G>C)	rs138700544	-	B/N/P	NA	Likely non-pathogenic
	*CHRNB2*	p.Phe472Ser (c.1415T>C)	rs200855905	4.118x10-5	PD/N/DC	NA	Likely non-pathogenic
17	*ADGRV1*	p.Glu2897Asp (c.8691A>C)	rs201586455	0.004302	PD/N/DC	NA	Likely non-pathogenic
**No segregation**							
18	*TBC1D24*	p.Glu94Lys (c.280G>A)	-	-	PD/D/DC	No	Benign
	*ADSL*	p.Asp215Asn (c.643G>A)	-	-	PD/D/DC	No	Benign
	*KCNMA1*	p.Val792Ile (c.2374G>A)	rs142770262	-	B/N/DC	No	Benign
19	*CACNA1H*	p.Ala2208Val (c.6623C>T)	rs777217625	-	B/N/P	No	VUS
	*EFHC1*	p.Arg42Pro (c.125G>C)	rs773598517	-	B/N/DC	No	VUS
	*ATP1A2*	p.Arg3His (c.8G>A)	rs781687346	8.12x10-5	PD/N/P	No	Likely non-pathogenic

NA: DNA not available from relatives. IP: Incomplete penetrance. CM: Human gene variation database code. MAF: Minor allele frequency in the ExAG (Last consulted May 2016). *In silico* predictors were PolyPhen2, Provean and Mutation Taster. N: Neutral; D: Deleterious; P: Polymorphism; DC: Disease causing; B: Benign; PD: Possibly damaging; VUS: Variant of uncertain significance.

*Variant detected in homozygous state.

^a^SUDEP cases sequenced in a previous custom resequencing panel.

After the pathogenicity score classification, 3 variants were classified as likely pathogenic, 20 variants were classified as unknown significance, 21 variants were classified as likely non-pathogenic and 12 variants were classified as benign variants for no segregation within the family.

Segregation studies were performed in 10 out of 19 SUDEP cases, and a total of 26 relatives were genetically analysed to ascertain familiar segregation. Four cases showed at least one variant with positive segregation (ID#1–4), four cases with variants with incomplete penetrance pattern (ID#5–8) and two cases with negative segregation (ID#18–19). Four subjects, previously investigated with inconclusive results[9], showed positive segregation (individuals ID#1, ID#3 and ID#4) or an incomplete pattern of inheritance (ID#5).

**Fig 2:**
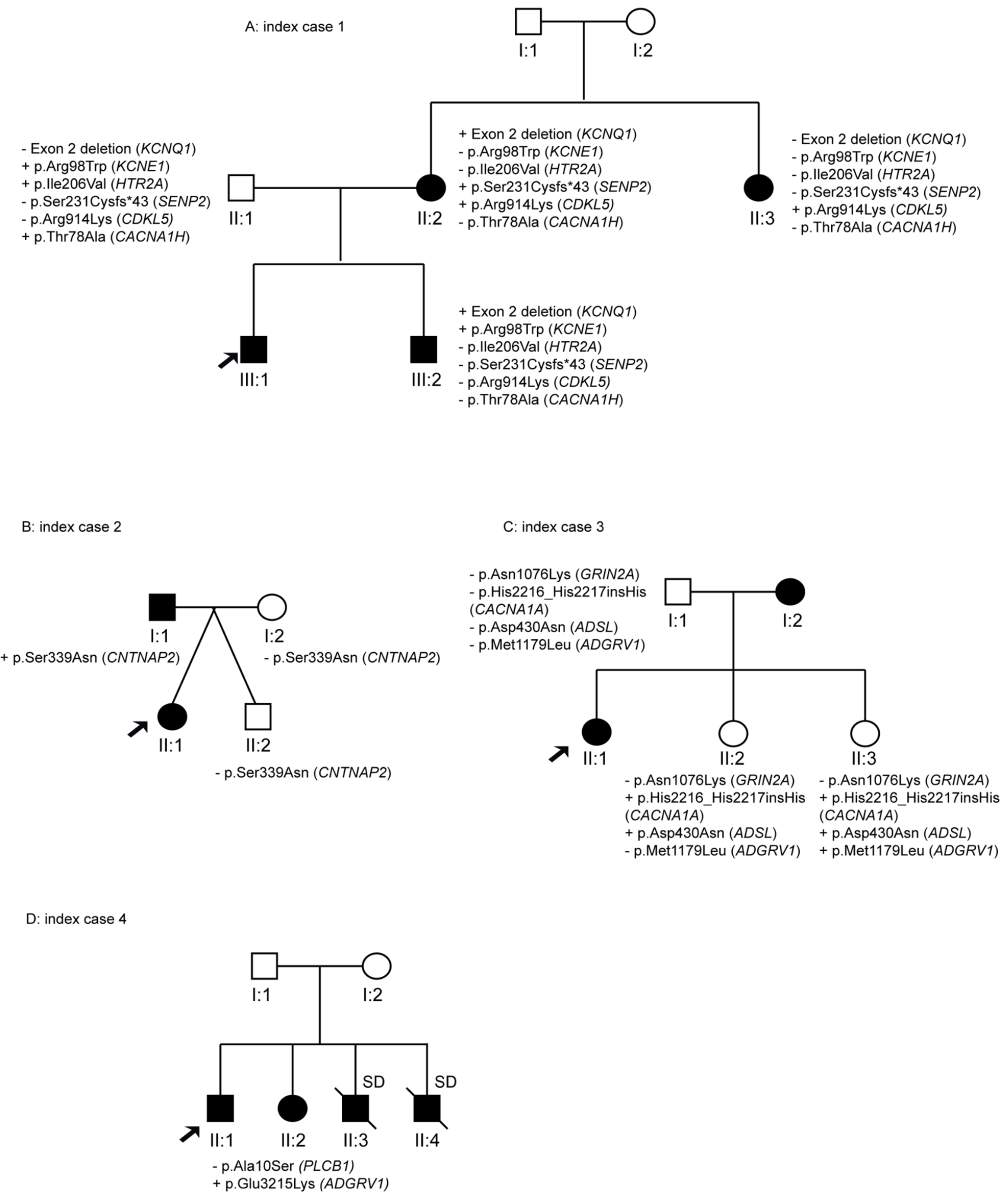
Pedigrees of the families with variants showing complete segregation (ID#1–4).

Genetic variants in *KCNQ1* and *CDKL5* genes were identified in ID#1, who had family members affected by epilepsy and/or long QT syndrome (Fig 2A). While the exon 2 deletion in *KCNQ1* (Fig 1) showed complete segregation with the LQTS, it is unlikely that the *CDKL5* variant was causative of the observed epilepsy, as mutations related to this gene typically occur *de novo* and are associated with early-onset, severe, drug-refractory epileptic encephalopathy [10].

The variant in the *CNTNAP2* gene identified in individual ID# 2 is a novel change classified as a variant of unknown significance. Segregation studies showed that the non-affected sister (II:2) and their parents (I:1 and I:2) did not carry this variant (Fig 2B). Heterozygous mutations in *CNTNAP2* have been identified in patients with a range of complex phenotypes, including intellectual disability, autism and schizophrenia [11, 12]. However, heterozygous CNTNAP2 mutations are also found in the normal population, and it is likely that this variant alone does not fully explain the patient’s phenotype.

Patient #3 is a 24-year-old woman with family history (individual I:2) of SUDEP. However, her mother was not available for genetic analysis. Segregation analysis in the non-affected sisters (individuals II:2, II:3) and the father (subject I:1) was inconclusive (Fig 2C).

Individual ID#4 is a 52-year-old man with family history of epilepsy (sister) and sudden death (his brothers). The variant identified in *ADGRV1* is shared by the affected sister (II:2), but DNA for the deceased brothers was unavailable (Fig 2D).

**Fig 3:**
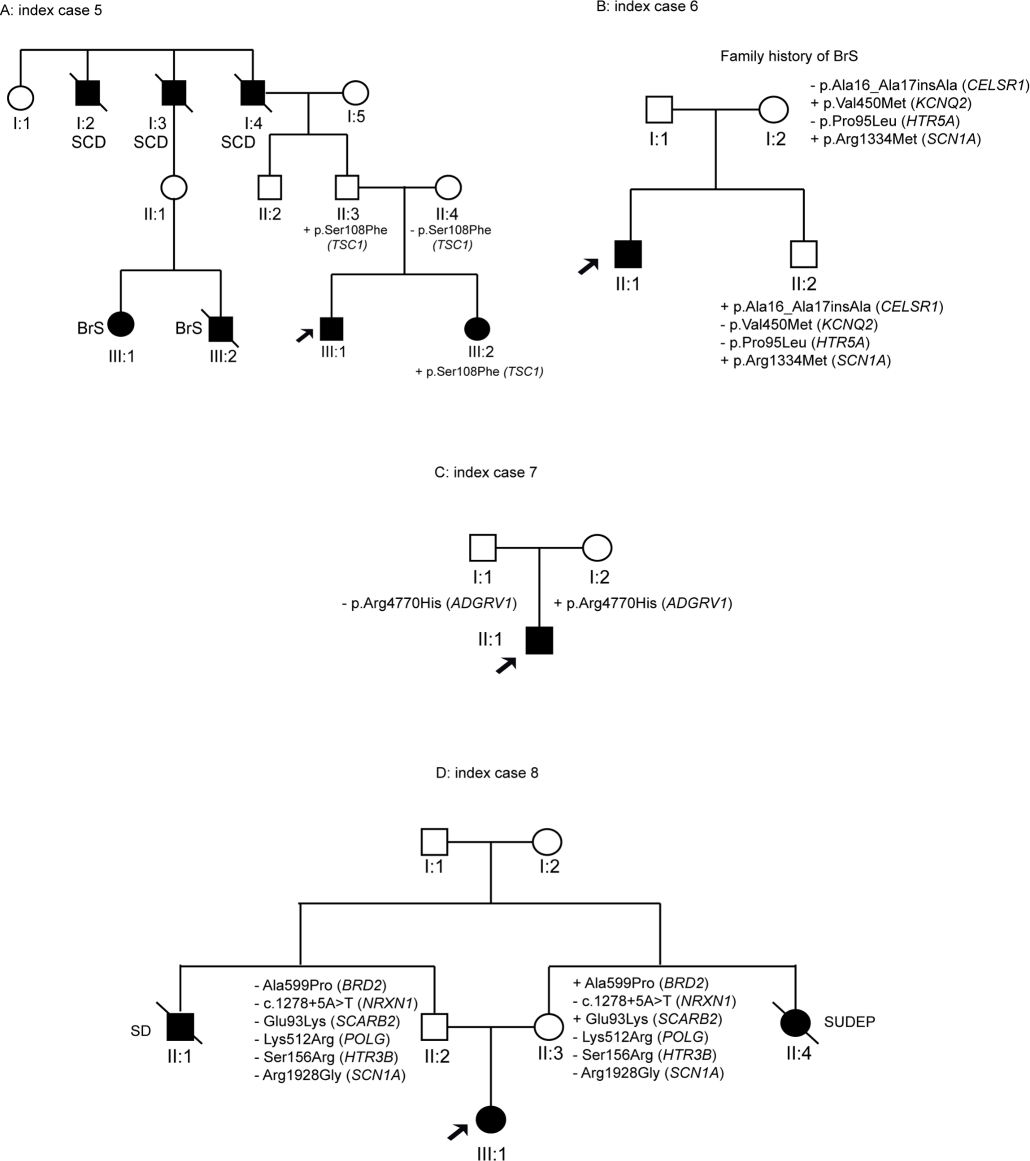
Pedigrees of the families with variants showing an incomplete pattern of inheritance (ID#5–8).

Individual ID#5 is a 15-year-old boy carrying a novel variant in the *TSC1* gene. This variant was shared by his healthy father (II:3) and affected sister (III:2) with epileptic encephalopathy. There were antecedents of SCD from the paternal lineage, and the father’s cousins (III:1 and III:2) were affected by Brugada syndrome (Fig 3A).

Another case with IP was ID#6, a 9-year-old boy affected by SUDEP and Brugada syndrome. The non-affected mother (I:2) had family history of Brugada syndrome and showed the variants in *KCNQ2* and *SCN1A* genes. The variant in the *SCN1A* gene was also present in the non-asymptomatic sister (II:2) (Fig 3B).

ID#7 is a 16-year-old boy affected by focal epilepsy and LQTS and carrying a rare genetic variant in *ADGRV1* that was harboured by his healthy mother (I:2) (Fig 3C).

Finally, ID#8 is a 21-year-old girl who suffered focal epilepsy and SUDEP. She carried six rare genetic variants in *BRD2*, *NRXN1*, *SCARB2*, *POLG*, *HTR3B*, and *SCN1A*. The brother’s father (II:1) died from sudden death and the mother’s sister (II:4) experienced SUDEP. Her healthy mother (II:3) carried the variants in the *BRD2* and *SCARB2* genes. Thus, segregation analysis in this family was also inconclusive (Fig 3D).

**Fig 4:**
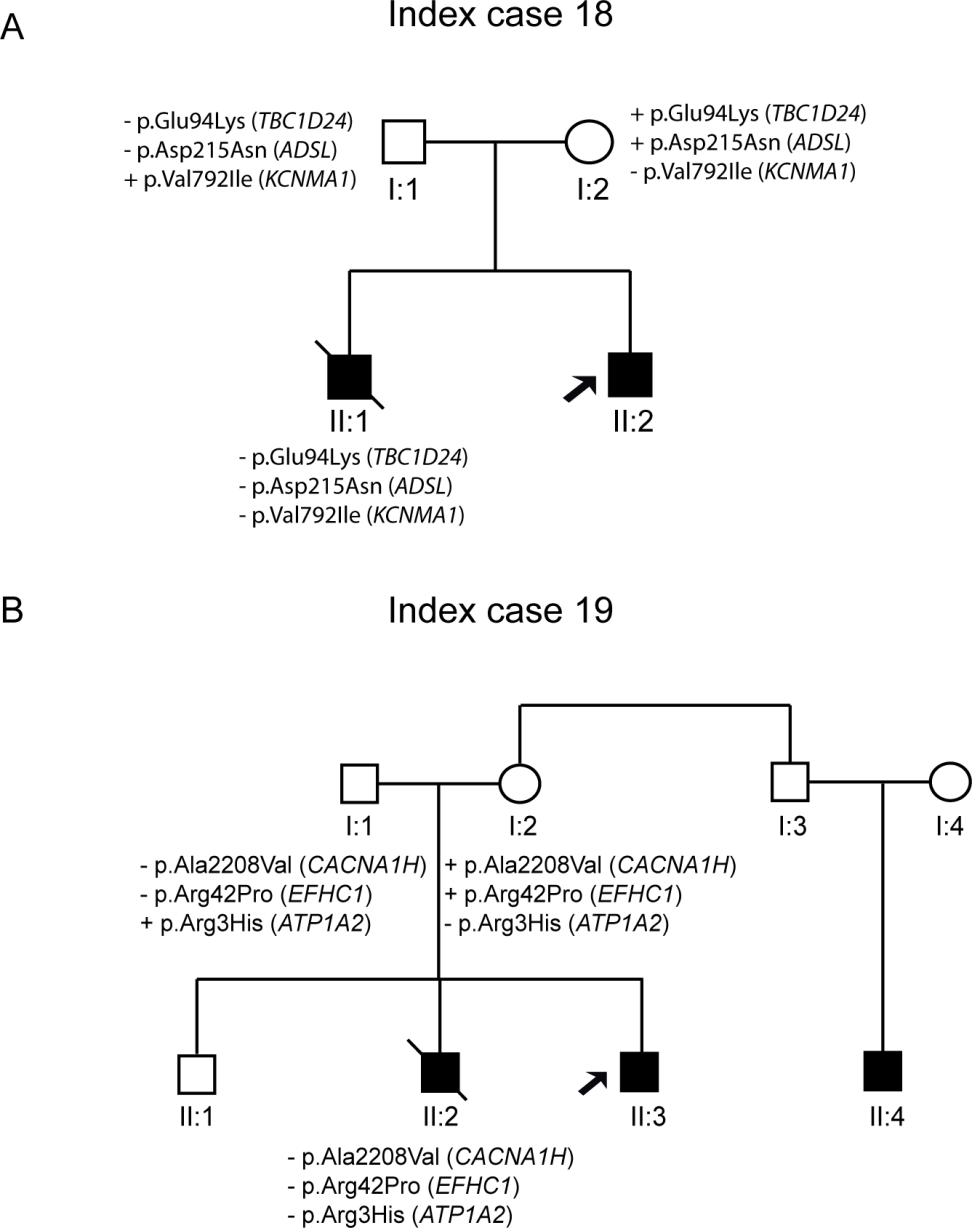
Pedigree of the family with variants that did not show segregation (ID#18–19).

There were two index cases with rare genetic variants not showing segregation with the pathology. ID#19 carried three variants in the *ADSL*, *TBC1D24*, and *KCNMA1* genes, but his dead brother (II:1) did not harbour any of these variants (Fig 4A). Patient ID#20 is a 12-year-old boy with focal epilepsy, and her brother (II:1) died of SUDEP while asleep at age 11 years. The maternal uncle has a 9-year-old child (II:3) who suffered a single seizure. Three rare genetic variants were identified in the index case, but only two of these variants (*EFHC1* and *CACNA1H*) were derived from the maternal lineage. However, these variants were absent in the deceased brother (II:2) (Fig 4B).

## Discussion

Sudden unexpected death is a catastrophic complication of human epilepsy with an incidence of 6.3–9.3 per 1,000 person years in epilepsy patients entering surgery programmes [1]. Despite recent advances, both cellular mechanisms and genetic bases remain largely unknown.

A leading hypothesis suggests a dysfunction of excitability that could underlie both epilepsy and cardiac arrhythmias, leading to death [13]. Recently, there has been increased interest in the potential association between epilepsy channelopathies and cardiac arrhythmias [5–7]. NGS is a fast and cost-effective method for the simultaneous detection of single nucleotide variants (SNVs) and small insertions or deletions (indels). Various studies have employed massive sequencing technology to identify new potential genes responsible for SUDEP[9, 14]. Olson et al. identified several CNVs with causative roles in some cases of paediatric epilepsy, suggesting that these types of variations may potentially be associated with SUDEP cases[15]. We performed a comprehensive genetic analysis using NGS technology to further explore the role of already known and novel candidate genes associated with epilepsy/SUDEP.

The first index case (ID#1) evidenced the coexistence of both LQTS and epilepsy. The exon 2 deletion showed positive segregation with the LQTS and the mutation in the *CDKL5* gene with the epilepsy in this family. Deletions in *KCNQ1* exons were previously reported by NGS in individuals with LQTS [16]. The *CDKL5* gene acts as a kinase, and one of its target proteins is MeCP2, which plays important roles in the function of nerve cells (neurons) and other brain cells and in the maintenance of connections (synapses) between neurons. Pathogenic variants in the *CDKL5* gene have been associated with a disorder known as X-linked infantile spasm syndrome, which is characterized by recurrent seizures called infantile spasms that begin in the first year of life and with intellectual disability; however, it is not clear how mutations in this gene can result in this phenotype [10].

In ID#2 we detected a novel rare variant in the *CNTNAP2* gene (contactin associated protein-like 2). Pathogenic alterations in this gene were associated with cortical dysplasia focal epilepsy syndrome, a rare disorder resulting in epileptic seizures, language regression, intellectual disability, hyperactivity and autism in nearly two-thirds of the patients[11]. However, variants in this gene have been found in control populations, whereas homozygous variants are rare in patient populations and have not been found in any unaffected individuals[12]. Although the father was affected by Brugada syndrome (BrS) and epilepsy and carried this variant, the absence of this complex phenotype and the lack of expression of the *CNTNAP2* gene in the heart complicate the association between this variant and SUDEP.

Individual ID#3 carried four rare genetic variants, and only variants with positive segregation were detected in the *GRIN2A* gene, although pathogenic score classified as probably no-pathogenic variant. Mutations in this gene have been associated with focal epilepsies as the phenotype of our index case.

The variant with positive segregation in the ID#4 was identified in the *ADGRV1* gene also known as *GPR98* (*MASS1* gene). The encoded protein contains a 7-transmembrane receptor domain, binds calcium and was expressed in the central nervous system. Pathogenic alterations in this gene have been associated with recurrent febrile seizures and afebrile seizures[17].

The variant identified in the ID#5 was in *TSC1* gene, which encodes for hamartin, a protein that interacts with tuberin and helps to control cell growth and size through the mTOR pathway[18]. Mutations in this gene are responsible for Tuberous sclerosis complex (TSC) and focal cortical dysplasia associated with drug-resistant epilepsy. Although the proband does not have a phenotype compatible with TSC, the role of hamartin deserves additional investigation due to its high expression in heart muscle [19].

The *KCNQ2* variant identified in individual ID#6 and the non-affected mother belongs to a large family of genes encoding potassium channels associated with benign neonatal seizures [20], but the role of this variant in the BrS phenotype of this family remains unclear. Conversely, *SCN1A* is a good gene candidate associated with a large range of epileptic phenotypes as well as cardiac dysfunction [21].

The variant identified in the *ADGRV1* gene of ID#7 is present in the non-affected mother, and no family history of epilepsy or LQTS is known. Mutations in this gene have been associated with Usher’s syndrome and febrile seizures familiar 4 (FEB4) and was not expressed in the heart, indicating that this variant was more related to epilepsy than to LQTS in this family[22]. Genetic testing of the main and associated genes related to LQTS would be the next strategy to identify the genetic cause of the disease in this patient.

The last genes with IP with the diseases were detected in ID#8. The non-affected mother carried variants in the *BRD2* and *SCARB2* genes, which could explain the SUDEP in her sister. Mutations in both genes have been associated with myoclonic epilepsy[23, 24]. *SCARB2* gene encodes for LIMP-2 protein, which is found in the heart, specifically in the intercalated discs, although its precise function is not clear[22].

Pathogenic variants in the genes identified in the present cohort were previously associated with syndromes involving intellectual disabilities with episodes of seizures difficulty the possible relation with SUDEP. However, ion channel genes, such as calcium (*GRIN2A)*, potassium (*KCNQ2*) or sodium (*SCN1A*) channels, may represent interesting candidate genes that underlie SUDEP, as the expression and regulation of these genes contributes to the central control of cardiac and respiratory function.

## Conclusion

Rare genetic alterations in genes encoding brain/heart ion channels are responsible for epilepsy associated with cardiac conduction disorder/SUDEP. NGS technology enables the comprehensive cost-effectives genetic analysis of all current known genes as well as candidate genes. Overall, we identified 5 rare genetic variants that segregated within the pedigree and explain the cardiac conduction disorder/SUDEP. Only one variant was a previously described gene, revealing four new potential genes associated with this entity. The new custom re-sequencing panel enabled the identification of potential pathogenic variants in three out of four negative SUDEP cases in a previous study. Genetic data should be interpreted by a group of experts, and translation into clinical and forensic fields should be done with caution.

## Methods

### Patients

The present study is a follow-up study in patients with clinical and EEG features consistent with a diagnosis of non-lesional (MRI-negative) focal or generalized epilepsy and a personal or family history of heart rhythm disturbances/cardiac arrhythmias/sudden death (Table 2).

**Table 2:** Clinical data for the investigated cohort.

Proband ID	Age (y)	Gender	Epilepsy type (onset, y)	Cardiac comorbidity	Tested family members (n)	Segregating variant(s)
1	32	Male	Focal epilepsy (24)	LQTS	4	2/6
2	31	Male	Focal epilepsy (21)	SUDEP	3	1/1
3	24	Female	Focal epilepsy (11)	SUDEP	3	1/4
4	52	Male	Focal epilepsy (43)	SUDEP	1	1/2
5	15	Male	Focal epilepsy (7)	SUDEP	3	0/1
6	9	Male	Generalized epilepsy (6)	SUDEP	2	0/4
7	16	Male	Focal epilepsy (11)	LQTS	2	0/1
8	21	Female	Focal epilepsy (17)	SUDEP	2	0/6
9	13	Female	Generalized epilepsy (12)	CPVT	NA	NA
10	26	Female	Focal epilepsy (26)	SUDEP	NA	NA
11	22	Female	Focal epilepsy (22)	SUDEP	NA	NA
12	25	Male	Generalized epilepsy (14)	SUDEP	NA	NA
13	21	Male	Generalized epilepsy (16)	SUDEP	NA	NA
14	13	Male	Generalized epilepsy (12)	SUDEP	NA	NA
15	19	Male	Focal epilepsy (17)	SUDEP	NA	NA
16	23	Female	Focal epilepsy (20)	SUDEP	NA	NA
17	17	Male	Generalized epilepsy (14)	SUDEP	NA	NA
18	23	Male	Focal epilepsy (20)	SUDEP	3	0/3
19	12	Male	Focal epilepsy (10)	BS	3	0/3
20	12	Male	Focal epilepsy (8)	SUDEP	NA	NA

NA: DNA not available from relatives. Familial individual tested excluding the proband. Variant/s segregated indicates the number of variants showing a complete segregation/total of the variants identified in the proband.

DNA samples of relatives were obtained when available to ascertain the segregation of each genetic variant. All patients received at least one surface electrocardiogram (EKG), which was evaluated by an expert cardiologist. Vague histories of heart rhythm disturbances in family members not clearly known by the proband or with any available clinical/instrumental data were excluded.

Each proband was genetically evaluated by target re-sequencing of exonic and intron-exon boundary regions. Clinical data and DNA from available relatives were obtained for testing in segregation studies of the rare genetic variants. The four SUDEP cases included in this cohort were genetically negative in a previous published SUDEP study [9].

All individuals and their family members signed a written informed consent to participate in the present study in accordance with the guidelines of the International Review Board of Catholic University. The study was approved through the Ethics Committee of Catholic University (Rome, Italy) in accordance with the principles outlined in the Declaration of Helsinki. The Full name of Ethic Committee is “Ethics Commitee, Università Cattolica del Sacro Cuore Largo Agostino Gemelli 8, 00168 Roma, Italy. The individuals in the present study provided written informed consent (as outlined in PLOS consent form) to publish these case details.

### DNA samples

Genomic DNA was extracted from whole blood or saliva with Chemagic MSM I, and the concentration was checked by fluorometry (Qubit, Life Technologies). DNA integrity was assessed on a 0.8% agarose gel.

### NGS studies

We designed a custom resequencing panel, which included 122 genes associated with epilepsy as well as the main genes associated with cardiac channelopathies (S1 Table). The final size was 487.874 kbp of encoding regions and UTR boundaries. We targeted one isoform for each gene and only coding exons plus 10 bp inside intronic regions. Exons were obtained from ensembl site[25] version 81 in GRCh38 and translated to hg19. Bait design was achieved using an in-house algorithm and submitted to Agilent SureSelect Design Web. The chip design was achieved using variable tailing bait and variable multiplicity bait to obtain homogeneous coverage and optimize sample load. The present bait design algorithm considers the GC content of the bait itself, and the target and surrounding region of the targeted gene. This algorithm also considers multimapping of the bait regions and uses, when available, previous NGS results.

Genomic DNA was fragmented by sonication using the Bioruptor (Diagenode). The 122 genes were enriched using the SureSelect Custom Target Enrichment System Kit (Agilent Technologies, Santa Clara, CA USA) according to the manufacture’s instructions for the "SureSelect Target Enrichment System for Illumina Paired-End Sequencing version B.1” (SureSelect XT Custom 0–499 kbp library, Agilent Technologies, Inc.). The paired-end sequencing process was developed on a MiSeq System (Illumina) using 2x76 bp reads length.

### Data analysis

NGS analysis was submitted to the GendiCall Pipeline (Gendicall Software from FerrerIncode), which cleans up and trims fastq files from sequencer and then Gendicall maps using either GEM[26] or BWA mapper[27]. Subsequently, bam was generated and sorted, and duplicates were removed with Picard (http://picard.sourceforge.net). Variant call for SNVs and Small Indels were performed using Samtools (v.1.3.1)[28] and internal Gendicall Caller. Annotation of called variants was based on several sources, and for population data, we used dbSNP[29], 1000 Genomes Project[30], Exome Variants Server (EVS)[31] and Exome Aggregation Consortium (ExAC) Cambridge, MA (URL: http://exac.broadinstitute.org). The protein predictors used in the present study were Polyphen2[32], Provean[33] and Mutation Taster[34]. Finally, we also used the splicing predictors MaxEntScan[35], FSPLICE[36], GeneSplicer[37] and NNsplice[38].

### Validation and bioinformatics analysis

The variants with MAF (minor allele frequency) lower than 0.1 (<1%) were assessed in locus-specific databases that include dbSNP[29], Ensembl genome browser[25], the 1000 Genomes Project[39], EVS (Exome Variant Server, NHLBI GO Exome Sequencing Project (ESP), Seattle, WA (URL: http://evs.gs.washington.edu/EVS/) [date (July, 2017) accessed] and ExAC[40]. Rare single nucleotide variants (SNPs) and insertions/deletions (indels) were confirmed by conventional Sanger sequencing, and copy number variants (CNVs) were confirmed by a multiplex ligation-dependent probe amplification (MLPA) technique.

Rare genetic variants and sequence regions with coverage lower than 30X were validated by the Sanger method. First, polymerase chain reaction (PCR) was performed, and the product was purified by ExoSAP-IT (USB Corporation, Cleveland, OH, USA) and directly sequenced by the dideoxy chain-termination method in ABI Prism Big Dye® Terminator v3.1 Cycle Sequencing Kit (Applied Biosystems, USA). Sequencing was processed in a 3130XL Genetic Analyzer (Applied Biosystems) and analysed by SeqScape Software v2.5 (Life Technologies), comparing the obtained results with the reference sequence from hg19. Protein numbering reflects the translation initiator methionine as +1.

The CNVs detected by NGS were confirmed by MLPA (MRC-Holland, Amsterdam, Netherlands) with the corresponding probe mix SALSA MLPA: SALSA MLPA P114 Long-QT probe mix and SALSA MLPA P297 Microdeletion Syndromes-2 probe mix. The MLPA DNA detection and quantification were performed according to the manufacturer’s protocol (MRC-Holland, Amsterdam, Netherlands). After the multiplex PCR reaction, electrophoresis was performed using the ABI3130xl Genetic Analyzer (Applied Biosystems, CA, USA), and the data were analysed with Coffalyser.net software (MRC-Holland). Significantly (30%) decreased or increased signals in the patient sample relative to controls were considered as deletions or duplications, respectively. Familial segregation of CNVs was also performed using MLPA. Variant frequencies (minor allele frequency, MAF>1%) were obtained from the ExAC collection (http://exac.broadinstitute.org/).

A pathogenicity score was assigned to each variant for classification into the following categories: pathogenic, likely pathogenic, variant of unknown significance, likely non-pathogenic and benign, as described*[41]*. *In silico* analysis of the pathogenicity of rare nonsynonymous variants was performed by Polyphen2, Provean and MutationTaster [42–44].

## Limitations

The present study has primary limitations. First, the lack of family segregation impedes the proper classification of the potential pathogenic role of each variant, and consequently, translation into clinical and forensic practice should be implemented with caution. In addition, functional in vivo/in vitro studies should also be performed to unravel the cellular mechanisms involved in SUDEP. We cannot exclude that these patients carry a genetic alteration in other genes that were not included in the present genetic panel. In these negative cases, and if the relatives are available, whole-exome sequencing studies could be a proper approach. Finally, additional studies in large cohorts should be performed to corroborate these results and identify new genetic alterations; however, the low incidence of SUDEP complicates sample collection.

## Supporting information

S1 Table List of genes included in the custom resequencing panel and the associated disease/s. (DOCX)
