# Supplementary material for: Targeted next-generation sequencing provides novel clues for associated epilepsy and cardiac conduction disorder/SUDEP
Source: PLoS One. 2017 Dec 19;12(12):e0189618. doi: 10.1371/journal.pone.0189618 (PMC5736193; doi:10.1371/journal.pone.0189618)
Supplement: S1 Table — (DOCX) [file pone.0189618.s001.docx]

| **Gene** | **NM Code** | **CCDS Code** | **Associated disease** |
| --- | --- | --- | --- |
| *ADGRV1 = GPR98* | NM_032119 | CCDS47246 | Generalized/myoclonic/absence epilepsies, febrile seizures |
| *ADSL* | NM_000026 | CCDS14001 | Epilepsy with mental retardation |
|  |  |  |  |
| *ALDH7A1* | NM_001182 | CCDS4137 | Pyridoxine dependent epilepsy/PNPO deficiency |
| *ARHGEF9* | NM_015185 | CCDS35315 | Early infantile epileptic encephalopathies and other predominantly focal or multifocal epilepsies |
| *ARX* | NM_139058 | CCDS14215 | Ohtahara syndrome, West syndrome, Lennox Gastaut syndrome, Early infantile epileptic encephalopathies |
| *ATP1A2* | NM_000702 | CCDS1196 | Familial hemiplegic migraine |
| *ATP6AP2* | NM_005765 | CCDS14252 | Epilepsy in X-linked mental retardation |
| *BRD2* | NM_001199455 | CCDS56420 | Generalized/myoclonic/absence epilepsies, febrile seizures |
| *CACNA1A* | NM_000068 | - | Lennox Gastaut syndrome, childhood/juvenile absence epilepsy, Familial hemiplegic migraine |
| *CACNA1C* | NM_000719 | CCDS44794 | Long QT syndrome, Brugada syndrome |
| *CACNA1H* | NM_021098 | CCDS45375 | Genetic generalized epilepsy, childhood absence epilepsy |
| *CACNA1I* | NM_021096 | CCDS46710 | Predominantly expressed in brain, involved in pacing neuronal firing and producing network oscillations |
|  |  |  | such as those that occurs during sleep and epilepsy (potential drug target) |
| *CACNB4* | NM_000726 | CCDS46426 | Genetic generalized epilepsy, juvenile myoclonic epilepsy |
| *CASR* | NM_000388 | CCDS3010 | Generalized/myoclonic/absence epilepsies, febrile seizures |
| *CDKL5* | NM_003159 | CCDS14186 | Ohtahara syndrome, West syndrome, Early infantile epileptic encephalopathy |
| *CELSR1* | NM_014246 | CCDS14076 | Congenital malformations of the central nervous system and adjacent structures related to defective |
|  |  |  | neural tube closure during the first trimester of pregnancy |
| *CHD2* | NM_001271 | CCDS10374 | Dravet syndrome, epilepsy of infancy with migrating focal seizures, Lennox Gastaut syndrome and other predominantly myoclonic epilepsies |
| *CHRNA2* | NM_000742 | CCDS6059 | Autosomal dominant nocturnal frontal lobe epilepsy |
| *CHRNA4* | NM_000744 | CCDS13517 | Autosomal dominant nocturnal frontal lobe epilepsy |
| *CHRNB2* | NM_000748 | CCDS1070 | Autosomal dominant nocturnal frontal lobe epilepsy |
| *CLCN2* | NM_004366 | CCDS3263 | Juvenile myoclonic epilepsy and genetic generalized epilepsy |
| *CLN3* | NM_001042432 | CCDS10632 | Batten disease |
| *CLN5* | NM_006493 | CCDS9456 | Batten disease |
| *CLN6* | NM_017882 | CCDS10227 | Neuronal ceroid lipofuscinosis |
| *CLN8* | NM_018941 | CCDS5956 | Neuronal ceroid lipofuscinosis |
| *CNTNAP2* | NM_014141 | CCDS5889 | Early infantile epileptic encephalopathies |
| *CSTB* | NM_000100 | CCDS13701 | Generalized/myoclonic/absence epilepsies, febrile seizures |
| *CTSD* | NM_001909 | CCDS7725 | Neuronal ceroid lipofuscinosis |
| *EFHC1* | NM_018100 | CCDS4942 | Juvenile absence epilepsy, juvenile myoclonic epilepsy and progressive myoclonic epilepsy (Lafora disease) |
| *EPM2A* | NM_005670 | CCDS5206 | Progressive myoclonic epilepsy (Lafora disease) |
| *FOXG1* | NM_005249 | CCDS9636 | West syndrome, other predominantly focal or multifocal epilepsies |
| *GABRA1* | NM_000806 | CCDS4357 | Dravet syndrome, Early infantile epileptic encephalopathy, Lennox Gastaut syndrome, Childhood absence epilepsy, Juvenile myoclonic epilepsy |
| *GABRB3* | NM_000814 | CCDS10019 | Lennox Gastaut syndrome, Childhood absence epilepsy |
| *GABRD* | NM_000815 | CCDS36 | Dravet syndrome, Febrile seizures plus and juvenile myoclonic epilepsy |
| *GABRG2* | NM_000816 | CCDS4358 | Dravet syndrome, Febrile seizures plus, Childhood/juvenile absence epilepsy |
| *GAD2* | NM_000818 | CCDS7149 | Diseases associated with GAD2 include autoimmune polyglandular syndrome type 2 and spastic cerebral palsy |
| *GAMT* | NM_000156 | CCDS12064 | Creatine disorders |
| *GATM* | NM_001482 | CCDS10122 | Creatine disorders |
| *GRIN2A* | NM_000833 | CCDS10539 | Photosensitive occipital lobe epilepsy, childhood epilepsy with centro-temporal spikes, epileptic encephalopathy with continuous spike-and-wave during sleep, Landau Kleffner syndrome, familial focal epilepsy |
| *GRIN2B* | NM_000834 | CCDS8662 | West syndrome, Lennox Gastaut syndrome, Familial focal epilepsy |
| *HCN1* | NM_021072 | CCDS3952 | Dravet syndrome, SUDEP |
| *HCN2* | NM_001194 | CCDS12035 | SUDEP |
| *HCN3* | NM_020897 | CCDS1108 | SUDEP |
| *HCN4* | NM_005477 | CCDS10248 | SUDEP |
| *HTR1A* | NM_000524 | CCDS34168 | Diseases associated with HTR1A include periodic fever / epilepsy |
| *HTR1B* | NM_000863 | CCDS4986 | Diseases associated with HTR1B include antisocial personality disorder / epilepsy |
| *HTR1E* | NM_000865 | CCDS5006 | Serotonin disorders |
| *HTR1F* | NM_000866 | CCDS2920 | Progressive myoclonus epilepsy |
| *HTR2A* | NM_000621 | CCDS9405 | Autosomal dominant lateral temporal lobe epilepsy |
| *HTR2B* | NM_000867 | CCDS2483 | Progressive myoclonus epilepsy |
| *HTR2C* | NM_000868 | CCDS14564 | SUDEP |
| *HTR3A* | NM_000869 | CCDS8365 | Migraine, epilepsy, obsessive-compulsive disorder, and affective disorder |
| *HTR3B* | NM_006028 | CCDS8364 | Diseases associated with HTR3B include gilles de la tourette syndrome / migraine / epilepsy |
| *HTR3C* | NM_130770 | CCDS3250 | Autism / epilepsy |
| *HTR3D* | NM_001163646 | CCDS54685 | Diseases associated with HTR3D include migraine / epilepsy |
| *HTR3E* | NM_182589 | CCDS3251 | Diseases associated with HTR3E include obstructive sleep apnea / Serotonin disorders / epilepsy |
| *HTR4* | NM_001040173 | CCDS34272 | Serotonin disorders |
| *HTR5A* | NM_024012 | CCDS5936 | Diseases associated with HTR5A include autistic disorder |
| *HTR6* | NM_000871 | CCDS197 | Diseases associated with HTR6 include schizophrenia |
| *HTR7* | NM_019859 | CCDS7408 | Diseases associated with HTR7 include autistic disorder |
| *KCNA1* | NM_000217 | CCDS8535 | Episodic Ataxia Type I, Episodic Ataxia with Myokymia, Hereditary Cerebellar Ataxia with Neuromyotonia |
| *KCNE1* | NM_000219 | CCDS13636 | SUDEP |
| *KCNE2* | NM_172201 | CCDS13635 | [Long QT Syndrome 6 and KCNE2-Related Familial Atrial Fibrillation](https://www.genetests.org/disorders/?disid=116533&ps=chld) |
| *KCNE3* | NM_005472 | CCDS8232 | Brugada syndrome |
| *KCNH2* | NM_000238 | CCDS5910 | SUDEP |
| *KCNJ11* | NM_000525 | CCDS31436 | Epilepsy/Seizure |
| *KCNMA1* | NM_002247 | CCDS7352 | Generalized/myoclonic/absence epilepsies, febrile seizures |
| *KCNQ1* | NM_000218 | CCDS7736 | SUDEP |
| *KCNQ2* | NM_172107 | CCDS13520 | Self-limited neonatal seizures and self-limited familial neonatal epilepsy, Ohtahara syndrome, Early infantile epileptic encephalopathy, epilepsy of infancy with migrating focal seizures |
| *KCNQ3* | NM_004519 | CCDS34943 | Self-limited neonatal seizures and self-limited familial neonatal epilepsy, epilepsy of infancy with migrating focal seizures |
| *KCTD7* | NM_153033 | CCDS5534 | Progressive myoclonic epilepsy |
| *LGI1* | NM_005097 | CCDS7431 | Autosomal dominant lateral temporal lobe epilepsy, Autosomal dominant epilepsy with auditory features |
| *MAPK10* | NM_002753 | CCDS43247 | Early infantile epileptic encephalopathies |
| *MBD5* | NM_018328 | CCDS33302 | Generalized/myoclonic/absence epilepsies, febrile seizures |
| *ME2* | NM_002396 | CCDS11948 | West syndrome, other predominantly focal or multifocal epilepsies |
| *MECP2* | NM_004992 | CCDS14741 | Lennox Gastaut syndrome |
| *MFSD8* | NM_152778 | CCDS3736 | Neuronal ceroid lipofuscinosis |
| *NHLRC1* | NM_198586 | CCDS4542 | Progressive myoclonic epilepsy (Lafora disease) |
| *NRXN1* | NM_001135659 | CCDS46282 | Pitt-Hopkings disease |
| *PCDH19* | NM_001184880 | CCDS55462 | Ohtahara syndrome, Dravet syndrome, Early infantile epileptic encephalopathy, Febrile seizures plus, Lennox Gastaut syndrome |
| *PHOX2B* | NM_003924 | CCDS3463 | Diseases associated with PHOX2B include neuroblastoma 2 and central hypoventilation syndrome, congenital |
| *PIGA* | NM_002641 | CCDS14165 | Early myoclonic encephalopathy |
| *PLCB1* | NM_015192 | CCDS13102 | Early infantile epileptic encephalopathy, epilepsy of infancy with migrating focal seizures |
| *PNKP* | NM_007254 | CCDS12783 | Neonatal epileptic encephalopathy, early myoclonic encephalopathy, other predominantly focal or multifocal epilepsies |
| *PNPO* | NM_018129 | CCDS11522 | Pyridoxine dependent epilepsy/PNPO deficiency |
| *POLG* | NM_002693 | CCDS10350 | Childhood occipital epilepsy, mitochondrial disorders |
| *PPT1* | NM_000310 | CCDS447 | Batten disease |
| *PRICKLE1* | NM_153026 | CCDS8742 | Progressive myoclonic epilepsy |
| *PRICKLE2* | NM_198859 | CCDS2902 | Progressive myoclonic epilepsy |
| *PRRT2* | NM_145239 | CCDS10654 | Self-limited familial and non-familial infantile epilepsy |
| *RNASEH2A* | NM_006397 | CCDS12282 | Early infantile epileptic encephalopathies |
| *RNASEH2B* | NM_024570 | CCDS9425 | Early infantile epileptic encephalopathies |
| *RNASEH2C* | NM_032193 | CCDS8111 | Early infantile epileptic encephalopathies |
| *RYR2* | NM_001035 | CCDS55691 | SUDEP |
| *SAMHD1* | NM_015474 | CCDS13288 | Early infantile epileptic encephalopathies |
| *SCARB2* | NM_005506 | CCDS3577 | Progressive myoclonic epilepsy |
| *SCN1A* | NM_001165963 | CCDS54413 | West syndrome, Dravet syndrome, Other predominantly myoclonic epilepsies, Early infantile epileptic encephalopathies, Epilepsy of infancy with migrating focal seizures, febrile seizures plus, SUDEP, epilepsy with myoclonic-atonic seizures |
| *SCN1B* | NM_001037 | CCDS12441 | Febrile seizures plus |
| *SCN2A* | NM_021007 | CCDS33314 | Self-limited familial and non-familial infantile epilepsy, Ohtahara syndrome, West syndrome, Dravet syndrome, Early infantile epileptic encephalopathy, Epilepsy of infancy with migrating focal seizures, Febrile seizures plus |
| *SCN3B* | NM_018400 | CCDS8442 | Long QT |
| *SCN4B* | NM_174934 | CCDS8389 | Long QT |
| *SCN5A* | NM_198056 | CCDS46796 | SUDEP |
| *SCN8A* | NM_014191 | CCDS44891 | Early infantile epileptic encephalopathy, Epilepsy of infancy with migrating focal seizures |
| *SCN9A* | NM_002977 | CCDS46441 | Dravet syndrome |
| *SENP2* | NM_021627 | CCDS33902 | SUMO1 |
| *SLC25A22* | NM_024698 | CCDS7715 | Early myoclonic encephalopathy, Ohtahara syndrome, Early infantile epileptic encephalopathy |
| *SLC2A1* | NM_006516 | CCDS477 | Epilepsy with myoclonic-atonic seizures, Childhood absence epilepsy, Genetic generalized epilepsy |
| *SLC9A6* | NM_006359 | CCDS14654 | Early infantile epileptic encephalopathies |
| *SPTAN1* | NM_001130438 | CCDS48036 | Ohtahara syndrome, West syndrome, Early infantile epileptic encephalopathy |
| *SRPX2* | NM_014467 | CCDS14471 | Epilepsy in X-linked mental retardation |
| *ST3GAL3* | NM_006279 | CCDS492 | Early infantile epileptic encephalopathy |
| *STXBP1* | NM_003165 | CCDS6874 | Early myoclonic encephalopathy, Ohtahara syndrome, West syndrome, Dravet syndrome, Early infantile epileptic encephalopathy |
| *SUMO1* | NM_003352 | CCDS2352 | Diseases associated with SUMO1 include orofacial cleft 10 and neuronal intranuclear inclusion disease |
| *SYN1* | NM_133499 | CCDS35233 | Diseases associated with SYN1 include epilepsy, x-linked, with variable learning disabilities and behavior |
|  |  |  | disorders and x-linked epilepsy - learning disabilities - behavior disorders |
| *TBC1D24* | NM_001199107 | CCDS55980 | Ohtahara syndrome, Early infantile epileptic encephalopathy, Epilepsy of infancy with migrating focal seizures, progressive myoclonic epilepsy |
| *TCF4* | NM_001083962 | CCDS42438 | Pitt-Hopkings disease |
| *TPP1* | NM_000391 | CCDS7770 | Batten disease |
| *TREX1* | NM_033629 | CCDS2769 | Early infantile epileptic encephalopathies |
| *TSC1* | NM_000368 | CCDS6956 | West syndrome |
| *TSC2* | NM_000548 | CCDS10458 | West syndrome |
| *UBE3A* | NM_130838 | CCDS32177 | Other predominantly myoclonic epilepsies |
| *ZEB2* | NM_014795 | CCDS2186 | Other predominantly focal and multifocal epilepsies |
